# Supplementary figures and images for: Effects of decreased Rac activity and malignant state on oral squamous cell carcinoma in vitro
Source: PLoS One. 2021 Jan 14;16(1):e0212323. doi: 10.1371/journal.pone.0212323 (PMC7808617; doi:10.1371/journal.pone.0212323)

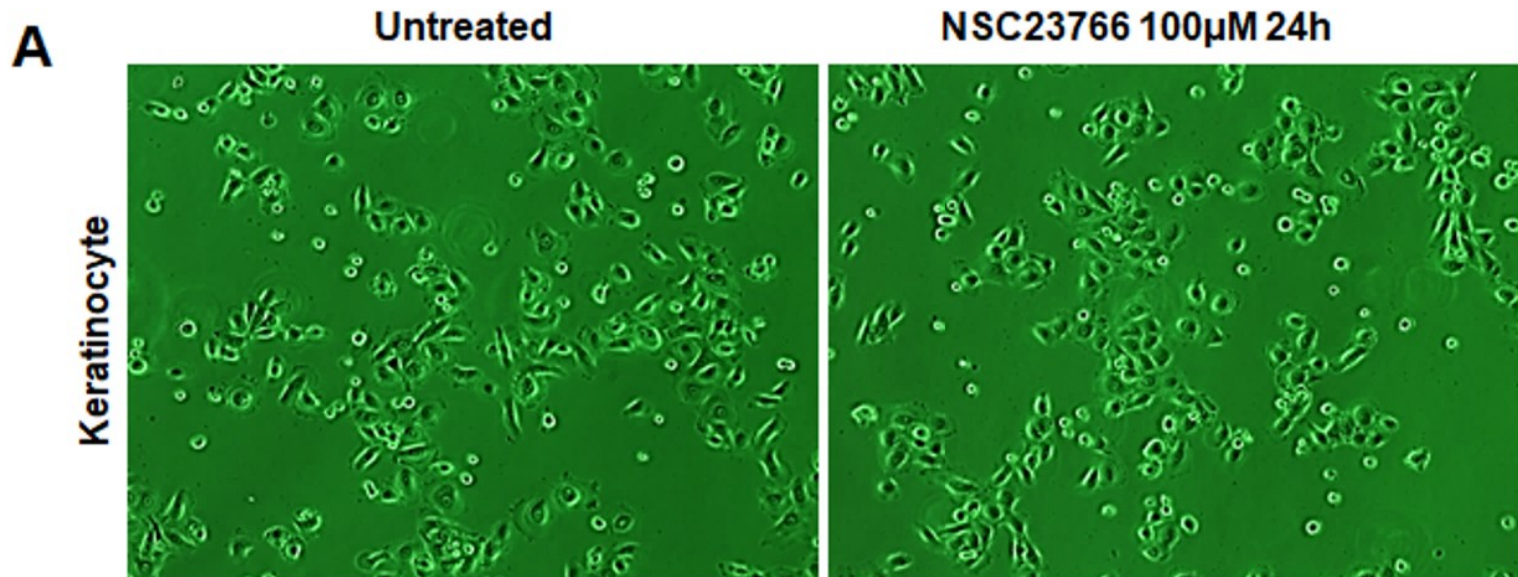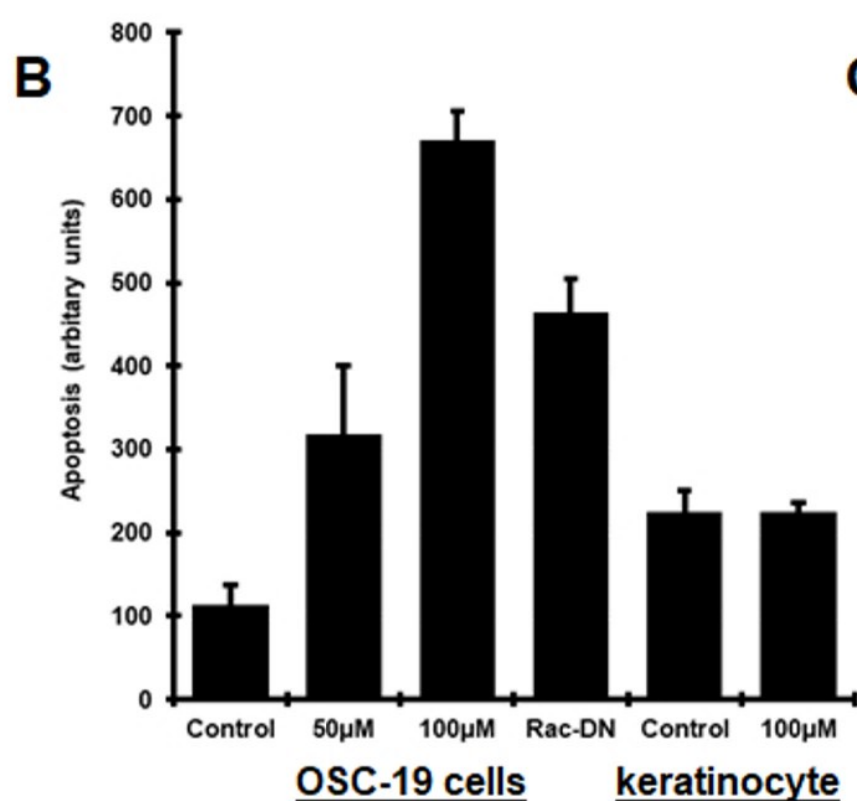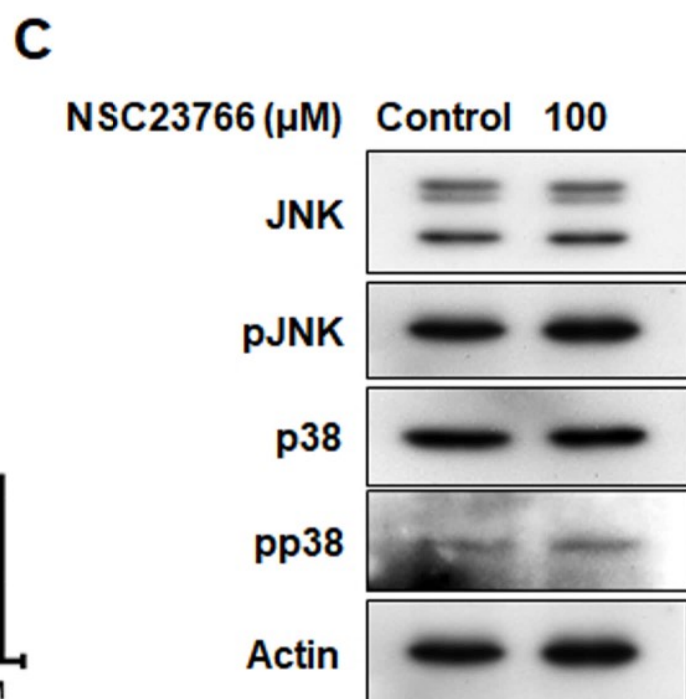

**Additional data**

Supplement: S1 Fig — (A) Phase-contrast micrographs demonstrating the effects of the selective Rac1 inhibitor NSC23766 on Keratinocyte cells. All cells were treated with 100 μM NSC23766 for 24 h. Keratinocytes did not demonstrate morphological changes. (B) A cell death detection ELISA assay was performed according to the manufacturer’s instructions to confirm the results regarding the apoptotic cell death. (C) Western blot analysis of JNK using antiJNK and pJNK polyclonal antibodies and a β-actin monoclonal antibody. Rac activity was inhibited by treating keratocytes with 100 μM selective Rac1 inhibitor NSC23766 for 9 h. (PDF) [file pone.0212323.s001.pdf]
